# Characterization of the complications associated with plasma exchange for thrombotic thrombocytopaenic purpura and related thrombotic microangiopathic anaemias: a single institution experience

**DOI:** 10.1111/vox.12090

**Published:** 2014-10-10

**Authors:** S McGuckin, J-P Westwood, H Webster, D Collier, D Leverett, M Scully

**Affiliations:** 1 University College London HospitalsLondon, UK; 2 University College LondonLondon, UK

**Keywords:** allergic reactions, central venous access, citrate reactions, plasma exchange, thrombotic thrombocytopaenic purpura, venous thrombotic events

## Abstract

**Background:**

Plasma exchange (PEX) is a life-saving therapeutic procedure in patients with thrombotic thrombocytopaenic purpura (TTP) and other thrombotic microangiopathic anaemias (TMAs). However, it may be associated with significant complications, exacerbating the morbidity and mortality in this patient group.

**Study Design and Methods:**

We reviewed all PEX procedures over a 72-month period, following the exclusive introduction of solvent–detergent double viral-inactivated plasma in high-volume users, such as TTP, in the United Kingdom (UK). We documented allergic reactions to plasma, citrate reactions, complications relating to central venous access insertion and venous thrombotic events (VTE) in 155 patient episodes and >2000 PEX procedures.

**Results:**

The overall complication rate was low. Allergic plasma reactions occurred in 6·45% of the cohort with only one episode of acute anaphylaxis. Similarly, VTEs were 6·45%, not significantly greater than in medical patients receiving thromboprophylaxis, despite added potential risk factors in TTP. Citrate reactions were the most frequent complication documented, but toxicity was significantly reduced by administration of further calcium infusions during the PEX procedure. There were no serious central line infections and no catheter thrombosis.

**Conclusion:**

Our data confirms that PEX continues to be a life-saving procedure in the acute TTP setting and, the procedure was not associated with an increased mortality and limited morbidity.

## Introduction

Thrombotic thrombocytopaenic purpura (TTP) is an acute life-threatening disorder associated with thrombocytopaenia, microangiopathic haemolytic anaemia and evidence of organ ischaemia. It results from a deficiency of the enzyme ADAMTS 13, important in cleavage of ultralarge von Willebrand factor multimers 1–3. Reduced ADAMTS 13 levels as in TTP are associated with the development of microvascular thrombi. Untreated, an acute presentation of TTP has an associated mortality of >90%. Therapeutic apheresis and the use of plasma have been the mainstay of treatment of acute TTP for nearly half a century and have had the greatest benefit on mortality outcomes. The benefit of PEX over plasma infusion has been demonstrated, but the mortality of acute TTP episodes remains between 10 and 20% 4.

Since 1 January 2006, the UK Department of Health have sourced non-UK, virally inactivated plasma for all children born after 1 January 1997 and all high-volume plasma users, including patients with TTP. For patients with TTP, solvent–detergent fresh-frozen plasma (SD-FFP), Octaplas (Octapharma, UK) a pooled plasma product that has undergone double viral inactivation is the preferred choice (DH Gateway reference 5999, 2006).

Although generally well tolerated, there are a series of potential complications associated with PEX. There have been a number of reports detailing adverse events related to line insertion, the plasma product and complications associated with it 5,6, resulting in death or morbidity in some cases. We have therefore undertaken a review of PEX procedures, plasma complications and adverse reactions because Octaplas has been exclusively used in the UK for patients with TTP. We have previously reported lower reaction rates with viral-inactivated plasma compared with standard fresh-frozen plasma (FFP)/cryosupernatant 7 and wanted to confirm this has remained consistent since exclusive use of Octaplas in this group of high-volume users.

### Methods

Within a single institution, we reviewed 155 sequential patient episodes [defined as inpatient admissions with acute presentation of thrombotic microangiopathic anaemias (TMA), specifically TTP] over a 72-month period, January 2006–December 2012. Our institution is a large tertiary referral centre for TTP but which also accepts and treats other thrombotic microangiopathies requiring treatment by therapeutic apheresis.

Cases were identified from hospital electronic records and case notes, and consent was given by all patients (MREC: 08/H0810/54 and MREC: 08/H0716/72). For all cases, we identified complications associated with our use of PEX. We recorded patient demographics, disease characteristics, laboratory parameters, red blood cell usage and the number of central venous access (CVA) lines inserted. Furthermore, we documented reactions to SD-FFP, citrate toxicities, venous thrombotic events (VTE) and the incidence of CVA-related line sepsis. Reactions to SD-FFP and ACD-A were classified and graded based on an adapted grading system 8.

Within our institution, all PEX procedures were carried out using centrifugation apheresis machines, either the COBE Spectra or Spectra Optia (Terumo BCT, Lakewood, CO, USA). The first PEX procedure routinely exchanges 1·5 plasma volumes as per BCSH guidelines 9. The volume of the replacement fluid required is calculated by multiplying the patients' weight by 60 ml for a 1·5 plasma volume and by 40 ml for a 1 plasma volume procedure.

We use ACD-A as anticoagulant for all PEX procedures. In an effort to minimize citrate reactions, it is local policy to administer calcium gluconate 2·2 mmol in 100 ml of normal saline over 15 min upon initiation of PEX, followed by calcium gluconate 0·04 mmol/kg/h for the remainder of the procedure.

All cases of VTE and their location are recorded following clinical assessment and vascular or radiological interventions. Day of PEX treatment, relation to CVA and platelet count were noted, as was initiation of appropriate therapy for the proven VTE.

In patients with CVA lines inserted, days *in situ* and subsequent line changes are closely monitored. When patients with TTP are first admitted, CVA lines are required for a variety of reasons, such as poor peripheral access and fluctuating neurological status. Femoral venous access is commonly obtained, as this is associated with reduced potential insertion risks and reduces the time delay to commence PEX.

However, femoral access is associated with a higher risk of infection 10. Femoral lines are replaced with neck (subclavian or internal jugular vein) lines within 48–72 h; minimizing infections is particularly important, as infections in patients with TTP can lead to a TTP exacerbation 7. The documented infections are highlighted, day diagnosed and bacteria isolated (Table6).

## Results

Over the 72-month period, a total of 2067 PEX procedures were performed in the 155 patient episodes, with each PEX procedure time ranging between 2 and 4 h. Patient demographics and admission characteristics are shown in Table1.

**Table 1:** Demographics and admission characteristics of patients treated with plasma exchange (PEX) for microangiopathic haemolytic anaemia/thrombotic microangiopathic anaemias

	Total *n* = 155
Female: Male	108:47 (2:1)
Mean age (years): Female (range)	43·9 (13–85)
Mean age (years): Male (range)	49·2 (19–87)
Ethnicity	
White	103
Afro/Caribbean	28
Other	24
Median presenting Hb, g/dl (range)	9 (4·8–15·1)
Median presenting platelets, ×10^9^/l (range)	16 (2–183[Table-fn tf1-1])
Median presenting LDH, IU (range)	1226 (227–5000)
Median length of stay, days (range)	14 (1–87)
Median number of PEX procedures (range)	11 (1–57)
Median number of transfused red cells: Females (range)	6 (0–26)
Median number of transfused red cells: Males (range)	6 (0–29)

Patient transferred from abroad-with thrombotic thrombocytopaenic purpura relapse-having been treated pre transfer and platelet count normal on return.

There were 108 females and 47 males presenting with acute TTP episodes, which is in keeping with 2:1 female preponderance. In total, 147 patient episodes were confirmed TTP (95%), of which 13 episodes (8%) were TTP relapses and 16 episodes (10%) a defined precipitant was identified, including HIV (*N *=* *7), pancreatitis (*n* = 3), congenital TTP (*N* = 6). The remaining cases were ‘other’ TMAs including aHUS or pregnancy-associated microangiopathies.

The overall risk of complications related to PEX is highlighted in Table2.

**Table 2:** Summary of complications associated with plasma exchange procedures

	Total (*N*)	Overall incidence (%)
Venous thrombotic events	10	6·45
Allergic plasma reactions	10	6·45
Citrate reactions	15	9·6
Line associated infections	13	8·3

### Incidence of VTE in acute TTP

Venous thrombotic events was documented in 10 of 155 (6·45%) patient episodes. These included six cases of pulmonary embolism (PE) and three cases of below-knee deep vein thrombosis (DVT) and one case of above-knee DVT. There were no episodes of line-associated thrombosis. Platelet count (Table3) on the day of documented PE ranged from 7 to 292 × 10^9^/l. The day of the documented PE ranged from Day 2 to 28. Platelet count on the day of documented DVT ranged from 86 to 243 × 10^9^/l, and they were diagnosed between days 6 and 12 from TTP diagnosis.

**Table 3:** Classification of position and platelet count when venous thrombotic events diagnosed

Event	Day of event	Platelet count (×10^9^/l)
BK DVT	6	243
	6	118
	8	100
AK DVT	12	86
PE	2	7
	3	77
	7	39
	13	62
	13	292
	28	148

BK DVT, below knee deep vein thrombosis; AK DVT, above knee deep vein thrombosis; PE, pulmonary embolus.

### Allergic reactions to SD-FFP

Within the cohort of 155 patient episodes, 10 (6·45%) plasma reactions were reported. Eight females and two males had plasma reactions (Tables4 and 5). Reactions included minimal signs and symptoms such as hives, itchy eyes to the more serious complications of chest, throat tightness to one major episode of stridor, which resulted in the administration of epinephrine and subsequent emergency transfer to the intensive care unit (ICU) for monitoring purposes. A full investigation excluded IgA deficiency or any other obvious cause for the reaction. A different batch of Octaplas was administered for all further PEX procedures, with premedication of chlorphenamine given with subsequent PEX procedures until remission without incident. We had no reported instances of transfusion-related associated lung injury or viral/infectious transmission.

**Table 4:** Classification of complications of therapeutic plasma exchange (PEX)

Grade of reaction	Effect of reaction	Plasma: manifestation and intervention	Citrate: manifestation and intervention
Grade 1: Mild	No medical intervention; procedure not delayed	Localised reaction (<25% skin affected): e.g. skin rash, hives: inlet rate reduced	Tingling, nausea: PO Ca^+^ followed by IV Ca^+^
Grade 2: Moderately severe	Not life threatening; medical intervention and/or delay of procedure required; no procedures terminated	Moderate reaction (>25% skin affected) e.g. skin rash, hives: IV bolus of Piriton and Hydrocortisone	Chest symptoms, (heaviness): IV Ca^+^ Gluconate, inlet rate reduced
Grade 3: Severe	Life threatening and/or procedure terminated	Anaphylaxis: IV bolus of Adrenalinine and nebulized salbutamol. Transfer to ITU	
Grade 4: Fatal	Death during or associated with PEX	Cardiopulmonary resuscitation	

ITU, Intensive therapy unit; IV, intravenous.

**Table 5:** Allergic reactions to plasma

Episode number	Sex	Type of reaction	Grading of reaction	Intervention
1	F	Facial itch	1	Inlet rate reduced
2	F	Facial itch, wheeze	2	Nebulized salbutamol
3	F	Hives	2	IV Piriton
4	F	Stridor	3	Adrenaline, H&P
5	F	Itchy eyes	1	Inlet rate reduced
6	F	Hives	2	IV Piriton
7	F	Lip swelling	2	H&P, nebulized salbutamol
8	F	Wheeze	2	H&P, nebulized salbutamol
9	M	Itchy eyes, chest Tightness	2	IV Piriton
10	M	Hives	2	IV Piriton

IV, Intravenous; H&P, hydrocortisone and piriton.

### Reaction to citrate

Within the cohort of 155 patient episodes reviewed, 15 (9·6%) had documented citrate (ACD-A) reactions, affecting 12 females and three males. The majority of ACD-A reactions were experienced by the patients during their initial PEX procedures, typically between days 1 and 4, with one patient experiencing reactions later in the course of their treatment (on D10 and D15). Symptoms experienced and reported by the patients ranged from: lip tingling (*n* = 5), pruritus (*n* = 2), nausea/lightheadedness (*n* = 6), chest and leg twitching (*n* = 2).

When reactions were reported by the patient during PEX, the inlet flow rate on the apheresis machine was initially lowered and if symptoms did not resolve, repeat boluses of 2·2 mmol of calcium gluconate IV were administered. However, no PEX procedures had to be permanently stopped.

### Complications related to central venous access

Central venous access is the preferred mode for PEX procedures for patients, as each PEX procedure can last between 2 and 4 h, therapy is required daily (or sometimes twice daily) and avoids the need for repeated venipuncture. In our cohort, there were a median of 11 PEX procedures per patient episode, range 1–57. However, 10·96% (*n* = 17) of the patient episodes had all PEX procedures carried out using peripheral access only.

Eighty-one femoral lines were initially sited upon admission compared with 55 neck lines. A total of 267 CVA lines were inserted for PEX for the remaining 138 patients requiring procedures. Of these, femoral lines (*n* = 94) were *in situ* for a median of 3·5 days (range 1–5 days) and neck lines, (*n* = 173) for a median of 8 days (range 1–10 days). All CVA lines were inserted either in an ICU setting by anaesthetists, theatre or in interventional radiology.

There were 13 (8·9%) documented episodes of line sepsis in seven female patients and four male patients (Table6). One female patient had two documented line infections: one with a femoral line sited on admission and a further episode with a subsequent neck line. One male had two documented line infections both associated with neck lines. All documented line sepsis episodes were treated with appropriate antibiotics and where possible, the line was replaced. There were no episodes of bleeding associated with line insertion, and no platelets were needed to cover any insertion procedure. Furthermore, there were no episodes of complications associated with line insertion such as pneumothorax or haemothorax.

**Table 6:** Central venous line related infections

Episode	Line site	Day of Sepsis	Organism identified
1	Neck	10	CNS
2	Femoral	5	*Escherichia coli*
3	Femoral	5	CNS
4	Femoral	2	CNS
5	Femoral	1	*Staphylococcus aureus*
6	Neck	4	*Staphylococcus aureus*
7	Femoral	4	CNS
8	Neck	7	CNS
9	Neck	2	CNS
10	Neck	8	CNS
11	Neck	1	*Enterococcus Faecalis*, Alpha-haemolytic *Streptococcus*, CNS
12	Neck	2	*Staphylococcus epidermis*
13	Neck	5	Acute Haemolytic *Streptococcus*

CNS, Coagulase negative staph.

### Red cell usage

Within our 155 patient episodes, a median of 6 units of red cells was transfused for both males (range 0–29 units) and females (range 0–26 units). The two patients who required 29 and 26 units of blood respectively, both had prolonged admissions which required intensive transfusion support over a period of several weeks.

## Discussion

Although PEX remains a life-saving therapeutic procedure in TTP and other TMAs 8, its use is associated with a series of potential complications. This review of therapeutic PEX procedures encompassed a large cohort of patients and examined complications of therapy. We have documented low rates of reactions to virally inactivated plasma SD-FFP (Octaplas), citrate reactions and complications relating to line infections, which may precipitate exacerbation of TTP. We have not had any patient deaths, either in this cohort or previously published, due to PEX or line insertion 7. Furthermore, the rate of VTEs is probably between 5 and 10% for symptomatic cases and not exclusively related to PEX, or the plasma used.

The incidence of VTE has not been highlighted specifically in the TTP literature. In our cohort, there were 10 cases of clinically diagnosed VTE, confirmed radiologically with an incidence of 6·45%. Although the overall risk of VTE may be perceived to be higher in patients with TTP, the risk for untreated medical patients is between 10 and 20%. Furthermore, using thromboprophylaxis (enoxaparin 40 mg s/c daily), the VTE rate in medical patients was reduced to 5·5% compared with 14·9% with placebo (no treatment) 11. Therefore, the rate in our cohort would be in keeping with medically treated patients. There has been a suggestion that VTEs could be precipitated by lower levels of protein S in Octaplas compared with standard FFP. However, review of the cases and follow-up of further procedures on comparing Octaplas with standard FFP, there was no greater risk, which has been reduced further by prophylactic measures subsequently introduced to prevent VTEs 7.

Within the UK, thromboprophylaxis has become a requirement for all eligible hospitalized patients. In patients with TTP, graduated elastic compression stockings (TEDs) are prescribed on admission. LMWH thromboprophylaxis, such as dalteparin 2500 IU subcutaneously BD, is administered once the platelet count is >50 × 10^9^/l. Within the presented cohort, VTE occurred at a range of platelet counts, including cases with low platelet counts (<10 × 10^9^/l) and in the normal range. Therefore, VTE could not be convincingly associated specifically with PEX and probably reflects multiple risk factors, including that TTP is a prothrombotic condition, immobility, use of steroids and patients’ weight.

With the sole administration of solvent–detergent fresh-frozen plasma, Octaplas, in therapeutic PEX, reactions, although still documented, are low in occurrence. There may have been underreporting of minor reactions; however, major reactions remain significantly reduced compared with data from standard FFP. Comparing two different plasma products in therapeutic exchange procedures, cryosupernatant was associated with reactions in 9·3% of PEX procedures, compared with 3·1% of those using Octaplas 7. A further review of 4857 PEX procedures from 509 patients documented 231 adverse reactions utilizing different replacement fluids. Nineteen patients, with thrombotic microangiopathy, FFP used in 594 therapeutic apheresis procedures, documented, 5 (0·1%) anaphylactic reactions 12. A comparison of standard FFP and cryosupernatant in patients with TTP suggests reactions occur in 65% of patients, which is unsurprising given the volumes required and length of time of therapy 13.

The Oklahoma group, in their latest review, reported 20 cases of plasma reactions out of a total number of 302 complications. Of the 20 cases, nine would be classified as grade 3, with anaphylaxis in one resulting in cardiac arrest, and fluid overload in another necessitating PEX being stopped and the patient intubated and ventilated. Furthermore, the authors suggested that reactions and complications have decreased with changes in management of patients, including less PEX to remission, with the use of adjunct immunosuppressive therapies 6.

Toxicity related to ACD-A anticoagulant solutions are frequently encountered. Modern apheresis equipment permits whole blood processing rates that are faster than those used in earlier case studies. This can inevitably increase the risk of citrate toxicity experienced by patients. Within the literature, it is reported that in a minority of PEX procedures, citrate toxicity can cause discomfort to patients and occasionally can lead to changes and abnormalities from baseline electrocardiographic readings 14. In our cohort, the majority of ACD-A reactions were experienced in initial PEX procedures. However, the overall rates are low with current calcium replacement protocols.

Utilization of CVA has increased dramatically in recent years. Whilst they are undoubtedly of extreme importance in the carrying out the PEX procedures, they represent a foreign body with direct access to the blood stream that can serve as a port of entry or a reservoir for infection. Central line blood stream infections are associated with high morbidity, mortality and cost 15.

Complications are reported within the literature with CVA and with associated infections. CVA was utilized in 23% of patients and two patients developed a pneumothorax as a direct result of CVA insertion 16. The Oklahoma group have reported seven deaths. Pulmonary haemorrhage leading to death occurred in three cases with death resulting from systemic infection in four cases. There were 22 episodes of thrombosis, and 17 resulted in catheter obstruction where catheters had to be removed 6.

Within our patient cohort, we have had no deaths, no haemothorax or pneumothorax and no line-associated thrombosis. Indeed, stricter adherence to our protocol of removing femoral CVA within 48 h of insertion and changing neck lines weekly would have reduced the infection rate further. This is not always possible as a patient's requirement for PEX outweighs line changes on some occasions. However, the benefit of this practice at reducing line infections has been demonstrated 7. Furthermore, line-insertion protocols, strict aseptic procedures and manipulation of lines using aseptic non-touch technique (ANTT) 17, have all contributed to the low infection rates.

In conclusion, we have presented data on the complication rates of therapeutic PEX primarily in patients with TTP, for whom treatment is intensive to achieve remission. Overall, the rates of complications are low, specifically for plasma reactions and VTEs. Line complications including sepsis have been reduced, and citrate reactions appear to be the most frequent event documented, with current infusion protocols. Therefore, therapeutic PEX appears to be a safe procedure, and the iatrogenic morbidity in this patient group has improved.
